# Disaster response self-efficacy and disaster preparedness levels of Turkish nurses: A cross-sectional study

**DOI:** 10.1186/s12873-025-01299-5

**Published:** 2025-08-20

**Authors:** Nilay Ercan Şahin, Türkan Karaca

**Affiliations:** 1 Nursing Faculty, Hacettepe University, Adnan Saygun Strett D-Buildings 1. Floor, Samanpazarı/Ankara, 06100 Türkiye; 2 Nursing Department, Health Science Faculty, Harran University, Osmanbey Campus, Central Classroom, Floor: 2, Şanlıurfa, Türkiye

**Keywords:** Disaster response self-efficacy, Disaster preparedness levels, Nurses

## Abstract

**Aim:**

This study was conducted to assess disaster response self-efficacy and disaster preparedness levels of Turkish nurses.

**Methods:**

This is a cross-sectional descriptive study involving a total of 195 nurses in Turkiye. A convenience sampling approach was used to reach nurses and the sampling criteria were full-time employment, being a university graduate, and voluntary participation. The data were collected online via a questionnaire created in Google Forms, using the Sociodemographic Information Form, the Disaster Preparedness Scale, and the Disaster Response Self-Efficacy Scale between September and December 2024.

**Results:**

The study revealed that nurses’ levels of disaster preparedness and disaster response self-efficacy were slightly above the maximum possible mean score. Significant differences were found between disaster preparedness and disaster response self-efficacy levels and factors such as gender, prior experience of a disaster, knowledge of the hospital’s emergency assembly area, and awareness of the hospital disaster plan. Furthermore, a moderate positive correlation was identified between disaster preparedness and disaster response self-efficacy levels.

**Conclusion:**

It was determined that nurses’ levels of disaster preparedness and disaster response self-efficacy were slightly above the maximum possible mean score. In line with these results, it could be suggested that disaster preparedness and self-efficacy in disaster response are associated with factors that enable nurses to intervene more effectively in disaster situations, highlighting the importance of training and awareness programs to ensure that nurses are adequately prepared for disasters.

## Introduction

Natural disasters, as well as man-made events are among the greatest threats faced by modern societies pose significant challenges to healthcare systems worldwide [1]. These events not only devastate physical infrastructure but also profoundly impact the psychological and social well-being of individuals and communities [2]. Nurses, being at the forefront of disaster response, are anticipated to take an active role and fulfill their responsibilities throughout all stages of disaster management, playing a crucial role in each phase [3]. This includes the pre-disaster phase, which involves implementing hospital disaster plans and identifying risks; the disaster phase, which focuses on delivering comprehensive care to victims, their families, and communities; and the post-disaster phase, which involves participating in recovery and reconstruction efforts. In this context, it is essential for nurses to possess core disaster nursing competencies, enabling them to be consistently prepared and confident in responding to disasters and providing effective care to those affected [4]. In order to provide the necessary care to those affected by a disaster, all nurses must be equipped with appropriate disaster response protocols and the professional skills required for their specific nursing roles [5]. Moreover, to effectively fulfill their roles in disaster response, it is crucial for nurses to have a strong sense of disaster response self-efficacy and maintain a high level of disaster preparedness.

A nurse’s ability to respond to disaster events is primarily influenced by their disaster response self-efficacy, which refers to nurses’ confidence in their ability to carry out tasks and make critical decisions during disaster situations [6, 7]. Moreover, a study found that disaster-related self-efficacy, along with prior experience in disaster management, plays a crucial role in shaping nurses’ disaster nursing competency [8]. Similarly, another study nurses had moderate knowledge, skills, and self-efficacy related to disasters [9].Furthermore, disaster preparedness among nurses is crucial for effective response to emergencies, yet evidence suggests nurses in countries at high risk of disasters had inadequate disaster knowledge and skills, leaving them ill-prepared to respond to such situations [10–14].

According to the World Risk Index 2022, Turkiye is among the most disaster-prone countries (Hilft) [15]. Turkiye frequently faces earthquakes, floods, landslides, wildfires, droughts, and urban fires [16]. The Kahramanmaraş-centered earthquakes on February 6, 2023, resulted in more than 50,000 fatalities and hundreds of thousands of injuries, highlighting Turkiye’s severe disaster risks [17]. Additionally, wildfires, floods, and landslides in recent years have displaced thousands and caused substantial economic losses. These recurring disasters highlight the urgent need for continuous evaluation of emergency response systems, policies, and procedures to strengthen the country’s healthcare capacity and disaster preparedness. This study was designed to determine nurses’ disaster preparedness and disaster response self-efficacy levels, their determinants, and the relationship between them. Considering Turkiye’s high disaster risk, it is striking that the levels of nurses’ disaster preparedness and disaster response self-efficacy have not been sufficiently evaluated. Therefore, this study was conducted to assess the levels of disaster preparedness and disaster response self-efficacy among nurses in Turkiye, explore their determinants, and examine the relationship between them. The findings of this study are expected to contribute to both national and international literature and provide guidance for future research. Consequently, the following research questions were identified:

Q1: What are the disaster preparedness and disaster response self-efficacy levels of Turkish nurses?

Q2: Is there a significant difference in disaster preparedness and disaster response self-efficacy levels between Turkish nurses?

Q3: Is disaster response self-efficacy positively correlated to disaster preparedness?

## Methods

### Research design

This research was conducted as a cross-sectional study to assess Turkish nurses’ disaster response self-efficacy and disaster preparedness levels.

### Participants

Nurses are considered a challenging group to recruit for research due to various barriers such as survey fatigue, hospital structures, and institutional review boards acting as gatekeepers to participant access [18–20]. Therefore, a convenience sampling method was used in this study. Initially, nursing colleagues and nursing department directors from hospitals known to the researchers were invited to participate and/or distribute the questionnaire. They were then asked to invite any nursing colleagues who might be interested in participating. The majority of participants originated from nurses working in cities such as Ankara, and Diyarbakır, though participants from various other provinces also contributed. While this sampling approach allowed for geographic diversity, it is important to note that the sample was not randomly selected, and institutional affiliations varied, including public hospitals, university hospitals, and private healthcare centers. The sampling criteria were full-time employment, being a university graduate, and voluntary participation. The study comprised 195 nurses. After data collection, the analysis was performed with 195 participants, and when using Cohen’s d = 0.5 (medium effect size) and α = 0.05, the study’s power was calculated to be approximately 93.5%. This indicates that the study has sufficient statistical power.

### Measurements

*Sociodemographic Information Form:* This form developed based on a literature review, comprising twelve items. This form collected data on nurses’ gender, age, marital status, education level, workplace, length of service, experience of disasters, involvement in disaster-related activities, knowledge of the hospital disaster plan, and the emergency assembly point [8, 9, 21–23]

*The Disaster Preparedness Scale (DPS):* This scale developed in Turkish language by Şentuna and Çakı in 2020, is designed to assess individuals’ preparedness for disasters. The scale consists of 13 items and 4 subscales (disaster physical protection (DPP), disaster planning (DP), disaster assistance(DA), and disaster warning systems (DWS)). The total score on the scale, which is rated using a four-point Likert type, ranges from 13 to 52. A higher total score indicates an increased level of disaster preparedness. The Cronbach’s alpha coefficient for the scale is 0.82 [24]. For the sample in this study, the Cronbach’s alpha coefficient was found to be 0.81.

*The Disaster Response Self-Efficacy Scale (DRSES):* This scale was developed by Li et al. in 2017 [7], and its Turkish validity and reliability study was conducted by Toraman et al. in 2018 [25]. The DRSES comprises 19 items and three sub-dimensions: Search And Rescue And Psychological Assessment Self-Efficacy (SRPA) (11 items), Disaster Assessment Self-efficacy (DAS) (4 items), and Adaptation self-efficacy (AS) (4 items). Participants rated each item on a 5-point Likert scale, with choices ranging from 1 (strongly disagree) to 5 (strongly agree). The total score is obtained by summing the responses, with higher scores indicating greater disaster response self-efficacy. In the Turkish validity and reliability study, the Cronbach’s alpha coefficient was found to be 0.96, demonstrating strong internal consistency and reliability. For the sample in this study, the Cronbach’s alpha coefficient was found to be 0.85.

### Data collection

The data were collected online between September and December 2024 through a questionnaire created in Google Forms. A convenience sampling approach was used to reach nurses based in Turkiye. The researchers distributed the survey through nurse colleagues and other relevant professional networks. The landing page of the survey presented a short overview of the study, followed by the participant information sheet and an option to give consent by selecting a checkbox. Electronic coding was used to prevent participants from completing the questionnaire for the second time.

### Data analysis

In the study, the statistical analyses were performed using the SPSS for Windows 25 statistical software.To assess the normality of the data distribution, Skewness and Kurtosis values, Kolmogorov-Smirnov significance value, Q–Q plot, Box-plot, and histogram were examined. Descriptive statistics were presented as mean, standard deviation, frequency, percentage, median, and minimum-maximum values. Independent Samples t-test was used to compare the means between two groups. One-way ANOVA was applied to compare means across more than two groups. Pearson correlation analysis was conducted to assess relationships between variables.

### Ethical considerations

The approval for the implementation of the study was obtained from the University of Ethics Committee for Social and Humanitarian Researches (Decison No:2024/528). In addition, participants completed the questionnaire after agreeing to complete the survey through the link provided. Participants provided electronic informed consent.

## Results

Table 1 shows data on the sociodemographic and work characteristics of the nurses. Of the participants, 82% were women, and 45% were between the ages of 26 and 35. Approximately 47% had 2 to 6 years of professional experience. While 68% had previously experienced a disaster, 34% had not received any training related to disaster preparedness.

**Table 1:** Nurses’ sociodemographic and work characteristics

Characteristics	n	%
**Gender**		
Female	161	82.6
Male	34	17.4
**Age**		
≤ 25	74	37.9
26-35	88	45.1
36-45	23	11.8
≥ 46	10	5.1
**Work seniority (year)**		
≤ 1	58	29.7
2-10	93	47.7
11-20	32	16.4
≥ 20	12	6.2
**Departments**		
Intensive care unit	84	33.8
Internal and surgery medicine	55	28.2
Emergency	29	14.9
Surgery	18	9.2
Other	26	13.3
**Prior disaster education/training experience**		
Yes	128	65.6
No	67	34.4
**Previous disaster experience**		
Yes	134	68.7
No	61	31.3
**Awareness of the hospital disaster plan**		
Yes	127	65.1
No	68	34.9
**Awareness of the emergency assembly point**		
Yes	139	71.3
No	56	28.7
Total	195	100.0

Table 2 presents the scores obtained from the DPS and DRSES and their subdimensions. The mean total score of the DPS among the participants was 35.37 ± 11.34, while the mean total score of the DRSES was 73.88 ± 15.11.

**Table 2:** Distribution of DPS and DRSES scores

	Mean	Std. Deviation	Minimum	Maximum
DPP	10.984	3.930	5.00	20.00
DP	6.7897	3.285	3.00	12.00
DA	9.128	2.726	3.00	12.00
DWS	4.328	1.933	2.00	8.00
**DPS Total Score**	35.3692	11.339	15.00	60.00
SRPA	38.266	8.773	17.00	50.00
DAS	19.194	4.120	8.00	25.00
AS	16.425	3.688	4.00	20.00
**DRSES Total Score**	73.887	15.110	34.00	95.00

Table 3 shows the distribution of DPS and DRSES scale scores according to certain characteristics of the nurses. Statistically significant differences were found between nurses’ gender, disaster experience, knowledge of hospital disaster plans, and awareness of emergency assembly points and DPS and DRSES scale scores. However, no statistically significant differences were found between age, years of work experience, or disaster-related training and DPS and DRSES scale scores.

**Table 3:** Distribution of scale scores according to the characteristics of the participants

	DPP	DP	DA	DWS	DPS (Total Score)	SRPA	DAS	AS	DRSES (Total Score)
**Gender**									
Female	10.69 ± 3.82	6.67 ± 3.24	9.04 ± 2.71	4.22 ± 1.88	34.57 ± 10.96	37.39 ± 8.63	18.82 ± 4.03	16.24 ± 3.68	72.47 ± 15.14
Male	12.35 ± 4.19	7.35 ± 3.45	9.50 ± 2.79	4.82 ± 2.09	39.14 ± 12.45	42.38 ± 7.10	20.94 ± 4.14	17.26 ± 3.63	80.58 ± 13.17
	t:-2.258 **p:.025**	t:-1.101 p:.272	t:-.875 p:.383	t:-1.652 p:.100	t:-2.158 **p:.032**	t:-3.075 **p:.002**	t:-2.76 **p:.006**	t:-1.464 p:.145	t:-2.90 **p:.004**
**Age**									
≤ 25	10.64 ± 4.17	6.74 ± 3.55	8.66 ± 2.83	4.60 ± 1.91	34.82 ± 12.00	38.28 ± 9.57	18.86 ± 4.17	16.85 ± 3.35	74.00 ± 15.49
26-35	11.10 ± 3.87	7.06 ± 3.55	9.55 ± 2.69	4.31 ± 1.90	36.21 ± 11.13	38.75 ± 8.09	19.55 ± 4.20	16.34 ± 3.68	74.64 ± 14.67
36-45	10.95 ± 2.97	6.17v2.34	8.78 ± 2.44	3.65 ± 1.82	33.30 ± 8.77	37.60 ± 8.59	19.17 ± 3.77	15.86 ± 4.63	72.65 ± 15.66
≥ 46	12.5 ± 4.60	6.10 ± 3.75	9.60 ± 2.36	3.90 ± 2.33	26.70 ± 13.88	35.40 ± 9.50	18.5 ± 3.77	15.30 ± 3.80	69.20 ± 16.13
	F:.699 p:.554	F:.628 p:.554	F: 1.687 p:.171	F:1.635 p:.183	F:.517 p:.671	F:.183 p:.694	F:.476 p:.700	F:.826 p:.481	F:.444 p:.722
**Work seniority (year)**									
≤ 1	10.98 ± 3.98	6.63 ± 2.92	8.81 ± 2.65	4.58 ± 1.91	35.15 ± 11.14	37.29 ± 8.70	18.41 ± 3.94	16.67 ± 3.14	72.37 ± 14.34
2-10	10.65 ± 4.08	6.88 ± 3.62	9.22 ± 2.87	4.32 ± 1.85	35.13 ± 11.79	39.39 ± 8.91	19.70 ± 4.34	16.75 ± 3.53	75.86 ± 15.46
11-20	11.50 ± 3.23	7.12 ± 2.77	9.12 ± 2.61	4.03 ± 2.03	36.06 ± 10.28	38.62 ± 8.13	19.28 ± 3.87	15.65 ± 4.60	73.56 ± 66.75
≥ 20	12.16 ± 4.19	5.91 ± 3.62	9.91 ± 2.19	3.91 ± 2.39	36.33 ± 12.61	33.25 ± 8.36	18.75 ± 3.59	14.75 ± 4.28	66.75 ± 14.85
	F:.759 p:.518	F:.455 p:.714	F:.634 p:.594	F:.775 p:.509	F:.087 p:.967	F: 2.115 p:.100	F: 1.234 p:.299	F: 1.636 p:.183	F: 1.634 p:.183
**Prior disaster education/training experience**									
Yes	10.83 ± 3.97	6.90 ± 3.28	6.90 ± 2.77	4.46 ± 1.96	35.59 ± 11.38	38.57 ± 8.77	19.38 ± 4.28	16.60 ± 3.53	74.55 ± 15.21
No	11.26 ± 3.85	6.56 ± 3.30	6.56 ± 2.64	4.05 ± 1.85	34.94 ± 11.32	37.68 ± 8.80	18.83 ± 3.79	16.08 ± 3.96	72.61 ± 15.93
	t:-.729 p:.479	t:.853 p:.496	t:.640 p:.523	t:1.407 p:.161	t:.381 p: 703	t:.667 p:.506	t:.880 p:.380	t:.920 p:.888	t:.852 p:.395
**Previous disaster experience**									
Yes	11.64 ± 3.80	7.42 ± 3.21	9.29 ± 2.70	4.59 ± 1.95	37.40 ± 10.97	39.55 ± 8.43	19.62 ± 3.88	16.82 ± 3.47	76.01 ± 14.28
No	9.54 ± 3.83	5.39 ± 3.01	8.75 ± 2.76	3.73 ± 1.75	30.90 ± 10.92	35.42 ± 8.90	18.24 ± 4.49	15.54 ± 4.00	69.21 ± 15.9
	t:3.563 **p:.000**	t:4.170 **p:.000**	t:1.295 p:.000	t:2.934 **p:.004**	t:3.842 **p:.000**	t:3.118 **p:.002**	t:2.191 **p:.030**	t:2.284 **p:.023**	t:2.973 **p:.003**
**Awareness of the hospital disaster plan**									
Yes	11.77 ± 4.030	7.35 ± 3.40	9.72 ± 2.52	4.80 ± 1.99	38.12 ± 11.43	40.55 ± 8.40	20.18 ± 3.91	17.00 ± 3.52	77.74 ± 14.51
No	9.51 ± 3.28	5.73 ± 2.77	8.01 ± 2.75	3.44 ± 1.45	30.22 ± 9.21	34.00 ± 7.85	17.35 ± 3.88	15.33 ± 3.76	66.69 ± 13.56
	t:3.964 **p:.000**	t: 3.365 **p:.001**	t:4.363 **p:.000**	t:4.966 **p:.000**	t:4.908 **p:.000**	t:5.305 **p:.000**	t:4.882 **p:.000**	t:3.077 **p:.002**	t:5.180 **p:.000**
**Awareness of the emergency assembly point**									
Yes	11.62 ± 3.99	7.28 ± 3.40	9.62 ± 2.58	4.65 ± 1.99	37.55 ± 11.30	39.75 ± 8.86	19.94 ± 4.01	16.61 ± 3.83	76.32 ± 15.51
No	9.39 ± 3.30	5.55 ± 2.61	7.89 ± 2.69	3.51 ± 1.48	29.94 ± 9.54	34.57 ± 7.41	17.32 ± 3.80	15.94 ± 3.27	67.83 ± 12.21
	t:3.705 **p:.000**	t: 3.426 **p:.001**	t:4.184 **p:.000**	t:3.845 **p:.000**	t:4.438 **p:.000**	t:3.865 **p:.000**	t:4.199 **p:.000**	t:1.152 **p:.251**	t:3.659 **p:.000**

Table 4 demonstrates the correlation between DPS subdimensions and total scores and DRSES subdimensions and total scores. A moderate positive relationship was found between the DPS total scores and DRSES total scores (r:.514, p:.000).

**Table 4:** Correlation between DPS and DRSES scores

	DPP	DP	DA	DWS	DPS	SRPA	DAS	AS	DRSES
DPP	1								
DP	.719**	1							
DA	.513**	.553**	1						
DWS	.601**	.578**	.419**	1					
DPS	.906**	.882**	.713**	.736**	1				
SRPA	.350**	.399**	.376**	.425**	.466**	1			
DAS	.438**	.474**	.599**	.411**	.577**	.706**	1		
AS	.266**	.286**	.367**	.264**	.351**	.781**	.631**	1	
DRSES	.388**	.431**	.471**	.424**	.514**	.964**	.837**	.870**	1

**Correlation is significant at the 0.01 level (2-tailed)

## Discussion

This study, conducted to evaluate disaster preparedness and disaster response self-efficacy among nurses, revealed that nurses’ levels of disaster preparedness and disaster response self-efficacy were slightly above the maximum possible mean score. Additionally, significant differences were found between disaster preparedness and disaster response self-efficacy levels and factors such as gender, prior experience of a disaster, knowledge of the hospital’s emergency assembly area, and awareness of the hospital disaster plan. Furthermore, a moderate positive correlation was identified between disaster preparedness and disaster response self-efficacy levels.

Nurses’ disaster response self-efficacy scores were found to be lower in some studies [21, 22, 26] compared to previous findings in the literature, with the exception of one study [9]. Based on the overall evaluation of these studies, it can be concluded that nurses’ disaster response self-efficacy is inadequate level. Similarly, studies have shown that nurses’ disaster preparedness levels are either moderate or low, and they were not completely ready to respond to disasters [12, 27, 28]. These findings highlight a critical gap in disaster preparedness among nurses, which may hinder their ability to respond effectively in emergency situations. Therefore, integrating comprehensive disaster training programs into nursing curricula and offering regular simulation exercises could be essential strategies for enhancing nurses’ readiness. Additionally, institutional policies that promote disaster preparedness, such as clearly defined roles, resource allocation, and psychological support mechanisms, may further improve nurses’ confidence and competence in managing disaster situations.

In this study, a significant difference was found between genders in terms of nurses’ disaster preparedness levels and disaster response self-efficacy, with male nurses scoring higher. Various studies in the literature have examined the relationship between nurses’ disaster preparedness levels, disaster response self-efficacy, and gender. For example, a study conducted by Wang et al (2023) reported that male nurses had a higher perceived self-efficacy in disaster preparedness [23]. Similarly, the other study found that male nurses students had higher disaster preparedness and disaster response self-efficacy [29]. This situation is thought to stem from women’s lower disaster preparedness and disaster response self-efficacy, which may be related to limited educational opportunities or societal gender roles. Therefore, more comprehensive research that takes into account cultural context is needed to better understand the impact of gender on disaster preparedness and response self-efficacy.

The current study, a significant difference was found between nurses with prior disaster experience and those without, with nurses who had disaster experience scoring higher in disaster preparedness and disaster response self-efficacy. This result suggests that nurses with previous disaster experience have gained more knowledge and skills, thus increasing their confidence in disaster preparedness and response. Similar findings are reported in the literature, studies found that nurse with disaster experience were better prepared for disasters [30] and reported higher self-efficacy [21]. In line with, it could be said that disaster experience provides nurses with the opportunity to gain real-time experience in disaster conditions, which can help them develop their practical knowledge and strategies.

In this study, a significant difference was found between nurses’ awareness of the hospital disaster plan and their knowledge of the emergency assembly point, with those who had higher awareness scoring better in disaster preparedness levels and disaster response self-efficacy. This result suggests that awareness of disaster plans and the emergency assembly point enhances nurses’ confidence in disaster preparedness and response, and that this awareness helps them act more effectively and consciously during disaster situations.

a moderate positive correlation was found between disaster preparedness levels and disaster response self-efficacy levels. This finding suggests that being well-prepared for disasters may enhance nurses’ confidence in responding during disaster situations. Preparedness equips nurses with the ability to act more effectively and consciously in disaster conditions, and this confidence can help them make more effective decisions during interventions.

### Limitations

This study was conducted with a sample of nurses from Turkiye, which limits the generalizability of the results. Data collection relied on self-reported measures, some nurses may have been reluctant to share their true situations or may have exaggerated their responses. Additionally, since the study was conducted online which may have restricted participation to nurses with internet access and digital literacy, potentially affecting the representativeness of the sample. Future research should consider longitudinal designs and larger, more diverse samples to provide a more comprehensive understanding of these relationships.

## Conclusion

In this study, it was determined that nurses’ levels of disaster preparedness and disaster response self-efficacy were slightly above the maximum possible mean score. Additionally, significant differences were found between disaster preparedness and disaster response self-efficacy levels and factors such as gender, prior disaster experience, knowledge of the hospital’s emergency assembly area, and awareness of the hospital’s disaster plan. Furthermore, a moderate positive correlation was identified between disaster preparedness levels and disaster response self-efficacy, indicating that the more prepared nurses are for disasters, the more confident they are and the more effectively they can respond in disaster situations.

### Implications for practice

The findings of this study suggest that improving nurses’ disaster preparedness and disaster response self-efficacy is crucial for enhancing their ability to respond effectively during emergency situations. Given the significant differences found between disaster preparedness and self-efficacy levels based on factors such as gender, prior disaster experience, and knowledge of the hospital’s disaster plan, targeted interventions are needed to address these areas.

Nursing education programs should emphasize the importance of disaster preparedness and equip nurses with the necessary skills and knowledge to feel confident in their ability to respond effectively in the event of a disaster. In addition, hospital administrators should ensure that all nurses are familiar with the hospital’s emergency protocols and assembly areas, as this knowledge is essential for quick and coordinated action during a disaster.

Moreover, adopting gender-sensitive approaches and tailoring the content and methods of training to meet the specific needs of female nurses are essential. Disaster training and confidence-building programs should be developed specifically for female nurses. In this way, all nurses will be better supported to respond more effectively and confidently in disaster situations.

Furthermore, promoting disaster preparedness training programs and simulation exercises could help nurses build their self-efficacy by allowing them to practice and refine their disaster response skills in a controlled environment. By enhancing both preparedness and self-efficacy, nurses will be better equipped to protect themselves, their patients, and the community during times of crisis.

## Data Availability

Data are available on request from the corresponding author.

## References

[1] Leider JP, DeBruin D, Reynolds N, Koch A, Seaberg J. Ethical guidance for disaster response, specifically around crisis standards of care: a systematic review. Am J Public Health. 2017;107(9):e1–e9. 28727521 10.2105/AJPH.2017.303882 PMC5551597

[2] Makwana N. Disaster and its impact on mental health: a narrative review. J Fam Med Prim Care. 2019;8(10):3090–95. 10.4103/jfmpc.jfmpc_893_19 PMC6857396 31742125

[3] Al Thobaity A, Plummer V, Williams B. What are the most common domains of the core competencies of disaster nursing? A scoping review. Int Emerg Nurs. 2017;31:64–71. 28029612 10.1016/j.ienj.2016.10.003 PMC7118449

[4] Al Harthi M, Al Thobaity A, Al Ahmari W, Almalki M. Challenges for nurses in disaster management: a scoping review. Risk Manag Healthc Policy. 2020;2627–34. 10.2147/RMHP.S279513 PMC7678497 33235533

[5] Banajah S. Critiquing disaster nursing competencies in relation to international standards writhing resilient health care system in Saudi Arabia. J US China Public Adm. 2018;15:181–97.

[6] Hammad KS, Arbon P, Gebbie K, Hutton A. Why a disaster is not just normal business ramped up: disaster response among ED nurses. Australas Emerg Care. 2018;21(1):36–41. 30998864 10.1016/j.aenj.2017.10.003

[7] Li H-Y, Bi R-X, Zhong Q-L. The development and psychometric testing of a disaster response self-efficacy scale among undergraduate nursing students. Nurse Educ Today. 2017;59:16–20. 28917131 10.1016/j.nedt.2017.07.009

[8] Mousavi M, Majidi A, Shabahang R, Besharat M, Bagheri Sheykhangafshe F, Samiee Z. The prediction of disaster nursing competency based on the self-efficacy and disaster-related experi-ences of nurses. J Clin Nurs Midwifery. 2020;9(1):582–90.

[9] Labrague LJ, Kamanyire JK, Achora S, Wesonga R, Malik A, Al Shaqsi S. Predictors of disaster response self-efficacy among nurses in Oman. Int J Disaster Risk Reduct. 2021;61:102300.

[10] Labrague LJ, Hammad K. Disaster preparedness among nurses in disaster-prone countries: a systematic review. Australas Emerg Care. 2024;27(2):88–96. 37778913 10.1016/j.auec.2023.09.002

[11] Labrague LJ, Hammad K, Gloe D, McEnroe-Petitte D, Fronda D, Obeidat A, Leocadio M, Cayaban A, Mirafuentes E. Disaster preparedness among nurses: a systematic review of literature. Int Nurs Rev. 2018;65(1):41–53. 28295314 10.1111/inr.12369

[12] Labrague LJ, Yboa BC, McEnroe–Petitte DM, Lobrino LR, Brennan MGB. Disaster preparedness in Philippine nurses. J Nurs Scholarsh. 2016;48(1):98–105. 26650188 10.1111/jnu.12186

[13] Öztekin SD, Larson EE, Akahoshi M, Öztekin İ. Japanese nurses’ perception of their preparedness for disasters: quantitative survey research on one prefecture in Japan. Japan J Nurs Sci. 2016;13(3):391–401. 26877076 10.1111/jjns.12121

[14] Tzeng W-C, Feng H-P, Cheng W-T, Lin C-H, Chiang L-C, Pai L, Lee C-L. Readiness of hospital nurses for disaster responses in Taiwan: a cross-sectional study. Nurse Educ Today. 2016;47:37–42. 26970707 10.1016/j.nedt.2016.02.025 PMC7131547

[15] Hilft BE. World Risk Report 2022. Ruhr University Bochum–Institute for International Law of Peace and Armed Conflict (IFHV); 2020.

[16] Benli H, Bacanlı M, Gündoğdu Ş, Yaman M, Esin M, Gökçe O, Karacameydan N, Kadirioğlu F, Kuterdem N, Zorbozan D. Türkiye’de afet yönetimi ve doğa kaynaklı afet istatistikleri. Ankara: AFAD; 2018.

[17] Gokceoglu C. 6 February 2023 Kahramanmaraş–Türkiye earthquakes: a general overview. Int Arch Photogramm Remote Sens Spatial Inf Sci. 2023;48:417–24.

[18] Broyles LM, Rodriguez KL, Price PA, Bayliss NK, Sevick MA. Overcoming barriers to the recruitment of nurses as participants in health care research. Qual Health Res. 2011;21(12):1705–18. 21844286 10.1177/1049732311417727

[19] Samia LW, Ellenbecker CH. Strategies to recruit difficult-to-reach home health care nurses for research. Appl Nurs Res. 2011;24(3):e17–e22. 20974091 10.1016/j.apnr.2010.02.002

[20] Weierbach FM, Glick DF, Fletcher K, Rowlands A, Lyder CH. Nursing research and participant recruitment: organizational challenges and strategies. JONA: J Nurs Adm. 2010;40(1):43–48. 10.1097/NNA.0b013e3181c97afb PMC2901155 20010377

[21] Hasan MK, Srijan MH, Mahatasim M, Anjum A, Abir AI, Masud MB, Tahsin S, Akram S, Shuvo MSR, Akter J. Factors influencing disaster response self-efficacy among registered nurses in Bangladesh. Prog Disaster Sci. 2024;100341.

[22] Vranada A, Anggriawan F, Wang T-J. Self-efficacy in disaster preparedness: insights from nurses in emergency and intensive care units. Media Keperawatan Indonesia. 2024;7(3):186–92.

[23] Ying W, Yu L, Mingfeng Y, Hui W, Chaohua P, Zhang P, Xinying N, Qu J, Changyan L. Disaster preparedness among nurses in China: a cross-sectional study. J Nurs Res. 2023;31(1):e255. 36469007 10.1097/jnr.0000000000000537

[24] Şentuna B, Çakı F. Balıkesir örnekleminde bir ölçek geliştirme çalışması: afet hazırbulunuşluk ölçeği. İdealkent. 2020;11(31):1959–83.

[25] Toraman AU, Korkmaz EK. Validity and reliability study of the Turkish adaptation of the Disaster Response Self-Efficacy Scale (DRSES). Disaster Med Public Health Prep. 2023;17:e297. 36789555 10.1017/dmp.2022.249

[26] Kuday AD, Erdoğan Ö. Relationship between emotional intelligence and disaster response self-efficacy: a comparative study in nurses. Int Emerg Nurs. 2023;70:101319. 37597280 10.1016/j.ienj.2023.101319

[27] Rizqillah AF, Suna J. Indonesian emergency nurses’ preparedness to respond to disaster: a descriptive survey. Australas Emerg Care. 2018;21(2):64–68. 30998877 10.1016/j.auec.2018.04.001

[28] Usher K, Mills J, West C, Casella E, Dorji P, Guo A, Koy V, Pego G, Phanpaseuth S, Phouthavong O. Cross-sectional survey of the disaster preparedness of nurses across the A sia–P acific region. Nurs Health Sci. 2015;17(4):434–43. 26245707 10.1111/nhs.12211

[29] İşleyen EK, Demirkaya Z. Relationship between disaster response self-efficacy and disaster preparedness in nursing students: after-earthquake study. Disaster Med Public Health Prep. 2024;18:e83. 38695197 10.1017/dmp.2024.69

[30] Lin C, Tzeng W, Chiang L, Lee M, Chiang S. Determinants of nurses’ readiness for disaster response: a cross-sectional study. Heliyon. 2023;9(10):e20579. 37810822 10.1016/j.heliyon.2023.e20579 PMC10550620

